# Development of a Sensory Method to Detect Food-Elicited Emotions Using Emotion-Color Association and Eye-Tracking

**DOI:** 10.3390/foods8060217

**Published:** 2019-06-18

**Authors:** Diana Ismael, Angelika Ploeger

**Affiliations:** Specialized Partnerships in Sustainable Food Systems and Food Sovereignty, University of Kassel, 34125 Kassel, Germany; a.ploeger@uni-kassel.de

**Keywords:** the color scale, emotion-color association (ECA), color-emotion association, eye-tracking, light colors, dark colors, general positive emotions (GPE), general negative emotions (GNE), sensory evaluation, self-reported questionnaire (SRQ), verbal emotion-based questionnaire (VEQ)

## Abstract

Studying consumers’ implicit emotions has been always described as a difficult and a complicated mission due to the emotions being of a non-cognitive nature. This research aims to develop a new method based on emotion-color association (ECA) to detect consumer’s implicit food-elicited emotions using an eye-tracker tool. The study was accomplished in two experiments. The first experiment intended to build a new color scale based on the emotion-color association using the eye-tracking method and a self-reported questionnaire (SRQ). The results showed that people tend to express their evoked positive emotions by choosing mostly the light colors, and favor to choose dark colors to reveal their evoked negative emotions. In the second experiment, a sensory evaluation was conducted employing the developed color scale in addition to verbal emotion-based questionnaire (VEQ) to detect the participants’ food-elicited emotions with different samples. The sensory evaluation consisted of taste, smell, and vision tests. The study demonstrated a consistency between the results of the verbal emotion questionnaire and the new color scale method. This consistency may refer to the capability of the developed scale, as a non-intrusive method that obtains prompt responses and avoids deliberate action, to rapidly detect the implicit emotions in a sensory evaluation for a better understanding of the consumer’s behavior.

## 1. Introduction

Emotions play a leading role in our food consumption behavior, which, in turn, affects our mood and generates food-elicited emotions. People spontaneously express their food-elicited emotions within their daily life activities [1,2,3,4,5,6]. Studying and measuring those emotions has received greater attention by sensory and consumer researchers [1,2,3,4,5,6] and various approaches have been developed to measure and understand these emotions [3]. Laros and Steenkamp [4] suggest a hierarchical approach for a better understanding of consumer emotions. The hierarchical model consists of three levels, which includes the superordinate level that is based on general positive emotion and general negative emotion, the basic level of emotions (anger, fear, sadness, shame, contentment, happiness, love, and pride), and the subordinate level that includes 41 emotion terms. 

However, emotions are known with their non-cognitive characteristic, which make them a difficult subject to measure [5]. According to Winkielman and Berridge [6], an unconscious emotion is considered impossible to report the moment it was evoked even though people’s behavior can reflect this emotion. Some of the emotion-measurement methods that provide conscious deliberate answers were criticized with being inefficient at detecting the real implicit emotions and the possibility of being affected by the Social Desirability Effect [7,8,9,10,11]. Thus, the need to develop new methods that are capable of measuring more implicit emotions has arisen.

Eye-tracking, which is a powerful technology that gives accurate data about the individual’s eye movement and fixation on a specific target, is considered to be a reliable method to capture the non-cognitive reaction [12,13]. Therefore, it obtains prompt responses without the need to write or talk. 

Moreover, colors carry intrinsic meanings and have a psychological relationship with emotions. Researchers suggest an innate functional relationship between colors and emotions [14]. The relationship between colors and emotions has two dimensions. The first one is the effect of colors on emotions, which is generally called color-emotion association. Several studies reported that light colors (white, pink, yellow, blue, purple, and green) evoke positive emotions like happiness, excitement, joy, and hope [15,16,17,18,19,20,21,22,23,24]. Other studies reported associations between dark colors (black, brown, and grey) and negative emotions (sadness, anxiety, fear, and boredom) [20,23,25,26,27]. The second dimension is how to express emotions using colors, which is called the emotion-color association (ECA). Two studies indicated that participants tend to express their emotion reactions using light and dark colors [28,29]. In those studies, participants used light color crayons such as yellow, orange, and green to color their drawing after watching happy scenes or hearing happy stories. While they used dark color crayons such as brown and black after listening to sad stories or watching sad scenes. Therefore, implicit positive and negative emotions can be expressed by new methods employing colors.

None of the currently used methods to study emotions have combined the eye-tracking technology with the emotion-color association concept to comprehend the implicit emotions on a superordinate level in sensory evaluations. This study aims to take advantage of the relationship between colors and emotions, using the ECA combined with the eye-tracking tool, to develop a new nonintrusive method in order to have a deeper insight on the implicit food-elicited emotions in a sensory test.

## 2. Materials and Methods 

**Figure 1:**
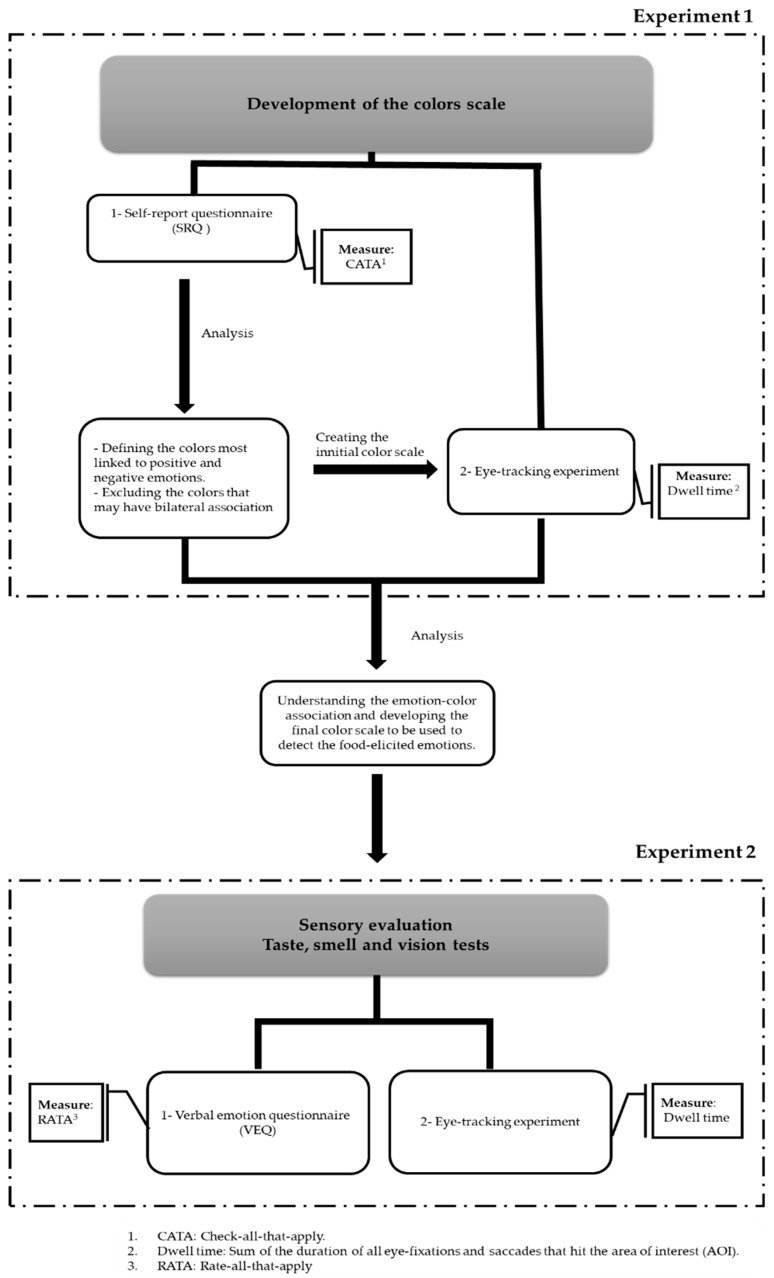
The above flowchart demonstrates the experimental design.

This study was conducted as a between-subject design in two experiments with a three-month interval and a total of 557 participants. As shown in Figure 1, the first experiment consisted of a self-reported questionnaire (SRQ) and a stationary eye-tracking experiment. The second experiment was a sensory evaluation that combined the eye-tracking method with a verbal emotion-based questionnaire (VEQ). Each experimental session was carried out individually. All the participants volunteered to take part in the current study, and each participant booked a date and time for an individual session via an online scheduling tool. Table 1 demonstrates the number, age, and gender of the participants in each experiment. 

### 2.1. First Experiment: Developing the Color Scale Based on the Emotion-Color Association

To study the emotion-color association, the current research used two types of measures. The first measure was a self-reported questionnaire that aimed to better understand the relationship between colors and emotions in order to develop the color scale. The second measure employed the developed color scale together with the eye-tracking tool to investigate the implicit emotion-color association.

#### 2.1.1. Self-Reported Questionnaire

A web-based questionnaire was designed using the online tool Typeform® and was carried out with 487 participants. The questionnaire consisted of two main parts. The first part of the questionnaire intended to investigate the color-emotion association and understand the perceived emotional reaction toward two sets of light and dark colors. The second part aimed to study the emotion-color association by detecting how participants may use light and dark color samples to express their evoked emotions. All the questions were of a check-all-that-apply (CATA) type.

#### 2.1.2. Procedures

**Figure 2:**
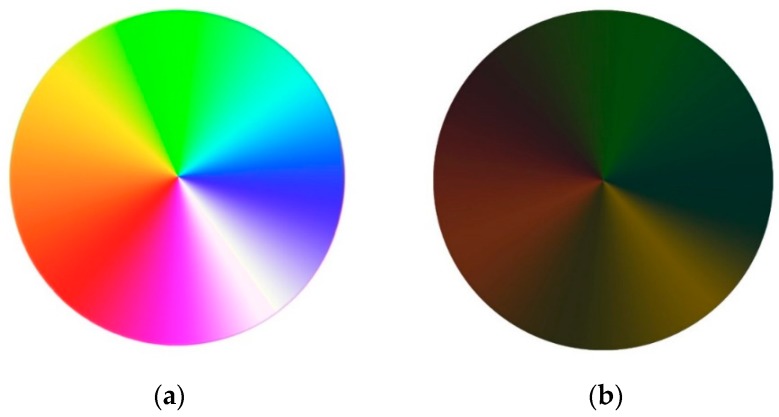
The color wheels used in the self-reported questionnaire (SRQ). (**a**) The wheel of the light colors, and (**b**) the wheel of the dark colors. Source: researcher’s own design.

In the first part of SRQ, two color wheels (Figure 2a,b), designed by the authors using Adobe Photoshop PS, were used to investigate the effects of light and dark colors on participants’ emotions.

The participants were first shown the light color wheel (LCW). Then they were asked to choose the term(s) that reflect their emotions toward the displayed color wheel. The question included a set of 22 positive and negative emotion-based terms (happy, hopeful, encouraged, motivated, peaceful, fulfilled, thrilled, enthusiastic, romantic, pleased, pride, angry, sad, frustrated, irritated, discontented, envious, scared, nervous, worried, guilty, and embarrassed) in addition to one neutral term “no emotion”. The emotion-based terms were chosen based on the subordinate level of the hierarchical model of consumers’ emotions [4]. A German translation based on the Food-Association Emotion lexicon was provided [30]. The same question was repeated using the dark color wheel (DCW) with the same set of emotion-based terms.

**Figure 3:**
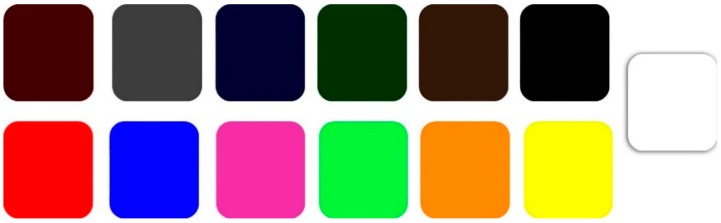
The displayed color samples that participants were asked to choose from in order to express their emotions after imagining a food consumption experience. Source: researcher’s own design.

The second part of the questionnaire investigated how participants may express their positive or negative emotions with light and dark color samples. A guided imagination technique was used to evoke the participants’ emotions [31,32,33]. The participants were first brought to imagine themselves consuming a specific food that evokes positive/negative emotion(s) (e.g., Take your time to imagine yourself eating food that evokes negative feelings within you, such as: irritated, guilty, or nervous). The two questions, which evoke the positive/negative emotions, were separated with a neutral question. The participants were asked to use one or more of the color samples (Figure 3) to express their evoked emotion(s). Then, participants were asked to choose the feature of the food that evoked the positive/negative emotion(s). The main provided features were: healthy, environmentally friendly, and tasty with items such as “The food which evoked positive feelings was healthy/ environmentally friendly, and tasty” and “The food which evoked negative feelings was tasty but neither healthy nor environmentally friendly”.

#### 2.1.3. Eye-Tracking Experiment

The eye-tracking experiment took place at the sensory lab at Kassel University, Germany and aimed at studying the implicit ECA. Emotional stimuli (pictures combined with emotional music) were displayed to provoke participants’ positive emotions (e.g., happiness, enthusiasm, and pleasant) or negative emotions (e.g., sadness, anger, and fear). The data were then recorded by using an individual laptop equipped with a specialized eye-tracker (SMI RED-250 mobile) manufactured by SensoMotoric Instruments (SMI, Germany). SMI RED-250 mobile is a fully portable, screen-based eye-tracker powered by USB and has a sampling rate of 60 Hz. All participants were confirmed to have normal or corrected vision. 

#### 2.1.4. Procedures 

**Figure A1:**
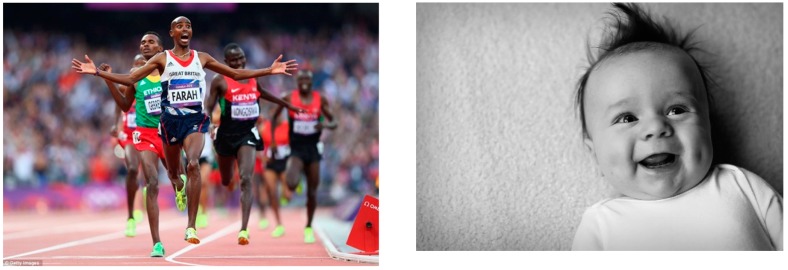
Positive emotional stimuli.

**Figure A2:**
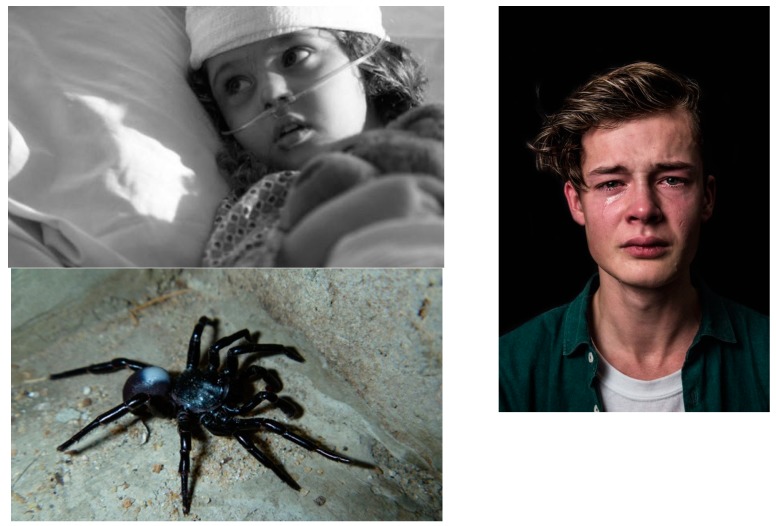
Negaive emotional stimuli.

A pre-test was performed to choose the most emotionally affective pictures for the trial. The pictures were rated on a 5-point scale (1 = very negative, 5 = very positive). Based on the pre-test, 20 emotional pictures were chosen to be included, together with emotional music, in the stimuli. Ten of the emotional stimuli (see Figure A1 and Figure A2) were positive and 10 were negative. Half of the 20 pictures were chosen randomly as chromatic and the other half as achromatic to study the effect of the pictures’ color on the participants’ choice between the dark and the light colors in the color scale.

Verbal and written explanation about the experiment and the content of the stimuli was provided. The participants were seated 50 cm in front of the laptop with the eye-tracker, and a simulation including detailed instruction was performed to accommodate the participants to the task. 

**Figure 4:**
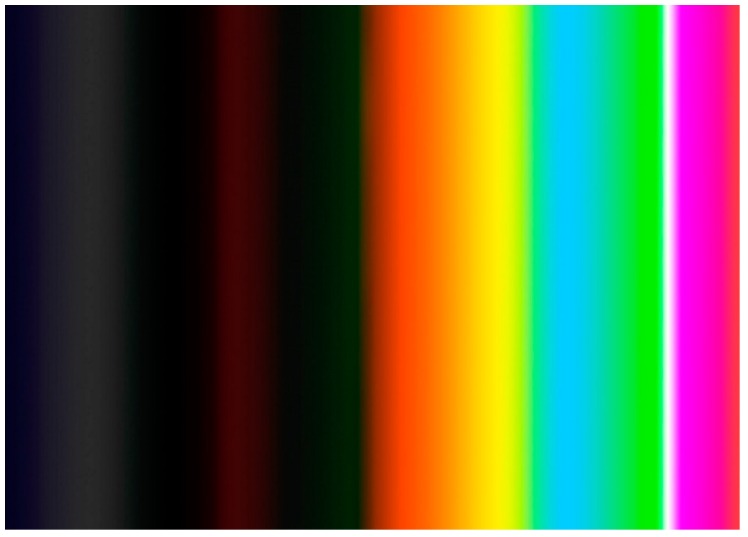
The color scale, including light color set and dark color set, that was displayed for 3 s after each emotional stimulus. Source: researcher’s own design.

**Figure A3:**
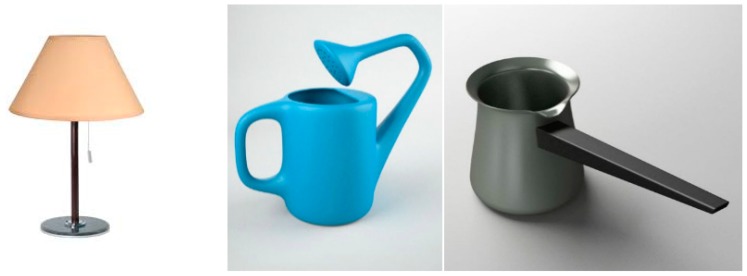
Neutral pictures.

The 20 stimuli were clustered into four groups. Two groups consisted of five positive emotional stimuli, and the other two consisted of five negative stimuli. The groups were displayed alternately based on the general emotions to avoid the bias that may result from the sequential display of positive/negative stimuli. After each group of stimuli, a neutral stimulus (see Figure A3) with no music was displayed for 10 s. Each emotional stimulus was displayed on the screen for 6 s [34]. A pre-examination showed that 6 s among other applied periods (10 s, 15 s, and 20 s) was the best period to leave the participants enough time to experience the evoked emotions without boring them with excessive time watching the stimulus. Since previous neuroscience and behavioral measures studies report that the response to an emotional stimulus happens in a matter of milliseconds (>1000 ms) [35,36], a panel of gradate colors, the color scale, were displayed for 3 s [37] after each stimulus. The color scale was designed using Adobe Photoshop PS and consisted of light and dark sets of colors (Figure 4). This method forces a time-pressure condition on participants and results in reducing the influence of conscious thinking. The participants were instructed to focus on the screen and fix their sight on the set of colors that express their emotion(s) at that moment. The position of dark and light sets of colors was randomly alternated in the color scale to avoid the bias resulting from keeping the color sets in the same position. A white fixation cross was presented on the center of each emotional photo in the last 500 ms prior to the offset of the stimulus [38] to correct the participants fixation tendencies and minimize the fixation bias that may be caused by the last eye-fixation position before displaying the color scale [39].

#### 2.1.5. Data Analysis

The data of the SRQ were analyzed following the standard procedures for CATA questions [40]. The non-qualified and incomplete answers were excluded. The frequency of use for each emotion term was determined by counting the number of participants that chose that term to describe their emotions toward each color wheel. Fisher’s exact test was carried out to identify significant differences between genders in each of the terms included on the CATA questions. Wilcoxon Signed-Rank Test was used to detect the significant differences between the general positive emotions (GPE) and the general negative emotions (GNE) for each color wheel. 

**Figure 5:**
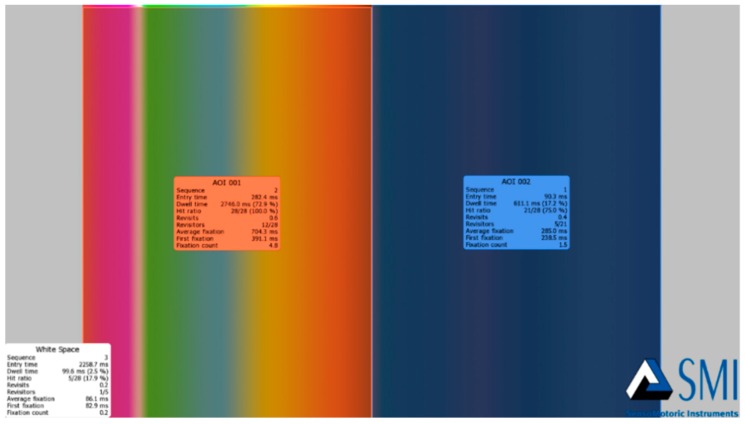
Key Performance Indication (executive summary). The AOI of the color scale after a positive emotional stimulus.

The eye-tracking data were collected using the Key Performance Indicator (KPI) in the BeGaze program. The color scale was divided into two Areas of Interest (AOI), and each set of colors was considered as an independent AOI. Dwell time value in milliseconds (ms), which represents the sum of the duration from all eye-fixations and saccades that hit the AOI, was collected. Figure 5 demonstrates the AOIs and the executive summary of the outcome data.

A paired t-test was conducted to detect any significant differences between dwell time on the AOIs after the positive and negative stimuli. Moreover, an analysis of variance was applied to investigate any differences between genders, stimuli colors, and the position of the dark and light side. 

All significance tests were done at a significance level of 5%. The Statistical Package for Social Sciences (IBM, SPSS Statistics, version 24) was used to analyze all collected data.

### 2.2. Second Experiment: Sensory Evaluation

The aim of the sensory evaluation was to examine the participants’ food-elicited emotions using the developed color scale and a verbal emotion-based questionnaire (VEQ). The sensory test took place in standard sensory booths at the sensory labs of Kassel University and Fulda University of Applied Sciences under artificial daylight type illumination, and temperature control (22–24 °C) with air circulation, according to ISO standards (ISO 8589:2007). The experiment consisted of taste, smell, and visual tests, respectively. All participants confirmed to have normal or corrected vision and no eating disorders or allergies for any of the samples.

#### 2.2.1. Samples

Eight samples were served in a sequential monadic testing. The samples were chosen to be acceptable by most people and easily available to consumers in supermarkets. The nature of the samples was declared in the announcement of the experiment to avoid allergy reaction to any of the samples. In the taste test, fresh apple (royal gala), ready-to-consume orange juice, and walnuts were served. Water was used for rinsing between samples. Oregano herbs, ground coffee, and fresh, red bell peppers were served in closed, odorless, plastic containers during the smell test, while the visual test consisted of a pear and a bottle of orange juice. 

#### 2.2.2. Procedures 

The participants were placed in the position that suits best the functionality of the eye-tracker. Then, they went under a simulation test to get familiar with the instructions and the new color scale. During taste, smell, and visual tests, the participants were asked to test the sample for around 20 s [41]. To guarantee the best period for the sample testing, the experimenter pretested different periods (15 s, 20 s, 40 s, and 60 s). The pre-test results verified the adequacy of the given 20 s time to test the samples and have an impression without getting bored. During the testing, a white screen was displayed on the monitor. Then a color scale appeared on a full screen for 5 s [12,42]. Participants were instructed clearly to look at the screen and use only their eyes to focus on the color set that expresses their food-elicited emotion(s). The participants who neither looked at the screen nor used the color scale had their data excluded. 

The position of the color scale was reversed randomly to avoid the bias responses’ result from displaying the same set of colors in the same position. To correct the participant’s fixation tendencies and minimize the fixation bias that may be caused by the last eye-fixation position, a white fixation-cross was presented on the center of the white screen [38] the last 2 s prior to displaying the color scale [39].

Subsequently, participants were asked to test the sample again, if desired, and rate their food-elicited emotion(s) using a verbal emotion-based questionnaire (VEQ) on a 5-point scale (1 = I do not feel it at all, 5 = I strongly feel it). Twelve emotion items were selected for the VEQ: happy, optimistic, proud, satisfied, encouraged, active, sad, regret, guilty, ashamed, scared, and angry. The terms were adopted from the Geneva Emotion Wheel (GEW), the Consumption Emotion Set (CES) [43], and the short-form of the Positive and Negative Affect Schedule (PANAS) [44]. All the questions were rate-all-that-apply (RATA) type.

The procedures were initially pretested with five participants (not included in the final data) to guarantee the best design of the experiment including participant’s seating position and given time to test the sample. The experimenter intended to start the session with a friendly conversation to relax the participants and avoid any kind of emotional upset or heavy pressure that prevents them from concentrating. 

#### 2.2.3. Data Analysis

For analytical purposes, the first six listed emotions in VEQ were considered positive emotions and the last six were considered as negative. The positive and the negative emotion terms were considered as Likert scale questions and the mean values of the items loading on the VEQ were computed for each participant and labelled as “general positive emotions (GPE)” and “general negative emotions (GNE)”. Then parametric analysis was conducted to detect statistical differences. 

Each set of colors was considered as an independent area of interest (AOI) and dwell time of each AOI was analyzed.

Analysis of variance was applied to analyze the differences between gender with regard to general emotions and dwell time. 

A paired t-test and a Wilcoxon signed-rank test were conducted to analyze the differences between GPE and GNE, and dwell time on the light colors versus the dwell time on the dark colors after testing each sample. 

## 3. Results

### 3.1. Self-Reported Questionnaire

In the first part of the SRQ, the participants were asked to choose the emotion-based terms they associate with two displayed color wheels (light color wheel and dark color wheel). The results showed a significant difference between GPE and GNE for each of the two wheels (*p* < 0.05). The LCW evoked more positive emotions, while more negative emotions were associated with DCW.

**Figure 6:**
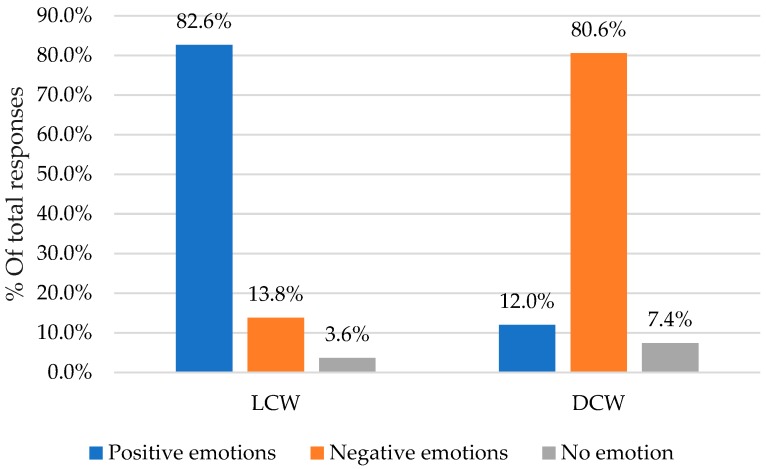
Percentages of total responses of positive, negative, and no emotion choices towards LCW and DCW. *n*= 487.

As shown in Figure 6, 82.6% of the total answers were positive emotion terms (happy, hopeful, encouraged, motivated, peaceful, fulfilled, and other), 13.8% of the answers were negative emotion terms (angry, sad, frustrated, irritated, discontented, envious, scared, and others) and 3.6% of the participants had no emotions towards LCW. When the participants were asked about DCW, 80.6% of the total answers were negative emotion terms, 12% of the answers were positive terms, and 7.4% had no emotions toward the colors. 

**Figure 7:**
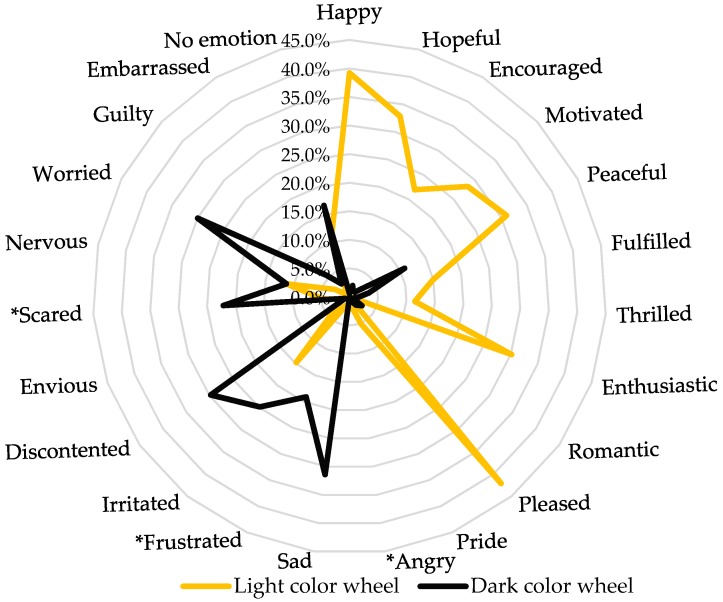
Percentage frequency of an emotional reaction given to each color wheel. Asterisks indicate significance results. *n* = 487.

Pleased, happy, and hopeful were the most commonly selected emotion-based terms when the participants were asked about their emotions toward LCW. Discontented, sad, and worried were the most selected terms when participants were asked about their emotions toward DCW (Figure 7). Results showed a gender impact with the terms “angry” (*p* = 0.007, 1.8% females and 6.8% males), “scared” (*p* = 0.014, 20% females and 31% males) and “frustrated” (*p* = 0.03, 21% females and 12% males) toward DCW. Gender had no impact on the effect of LCW on emotions (*ps* > 0.05).

The second part of SRQ aimed to investigate how the participants would use the colors to express their evoked emotion(s) by imagining a food experience consumption. The frequency of use of orange, yellow, and green to express the evoked positive emotion(s) were the highest (53%, 37%, and 36%, respectively). On the other hand, 42%, 38%, and 34% of the participants chose grey, brown, and black, respectively, to express their negative evoked emotion(s). Red was one of the first five colors used to express both positive and negative emotion(s) (26.5% and 20.5%, respectively). The results showed that the gender had an impact only with regard to the white sample (*p* < 0.05) in which females chose ‘’white” more than males to express their positive emotion(s).

**Figure 8:**
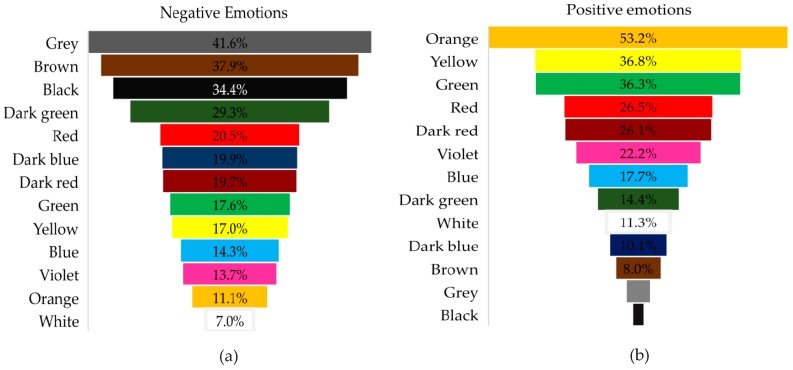
Percentage frequency of color choices to express the evoked emotion(s). (**a**) The color choices to express negative emotions. (**b**) The color choice to express positive emotions.

Figure 8a,b illustrates in order, the most and the least chosen colors to express positive and negative emotion(s). 

Orange was the color chosen with the greatest frequency to express positive emotion(s), while black was the least. On the contrary, grey was chosen with the greatest frequency after evoking negative emotion(s), while white was the least.

#### The Characteristics of the Food in the Imagined Consumption Experience 

After the participants were asked to imagine eating a specific food that provokes positive/negative emotions, they were instructed to describe that food by choosing one or more of three aspects: health, environmental impact, and taste. “Healthy, environmentally-friendly, and tasty” was the first feature chosen by three-quarters of the participants to describe the food that evoked their positive emotion(s). In addition, 45% of the participants chose “only tasty”, whereas 5% chose “healthy but not tasty” and only 2% of the participants declared that “environmentally-friendly but not tasty” food makes them have a positive emotion(s). 

Furthermore, 51% of the participants declared that, if the food is “neither healthy nor environmentally-friendly, yet tasty”, the food provokes a negative emotion(s). A very close percentage of participants (48%) showed that if the food is “healthy but not tasty”, it provokes negative emotion(s). In addition, 40% of the participants reported that if the food is “environmentally-friendly but not tasty”, it provokes a negative emotion(s). Table 2 demonstrates the frequency of participants’ choices to describe the food in each situation. 

### 3.2. Eye-Tracking Experiment

The results showed a statistically significant difference between dwell time on the light color set (LCS) and dwell time on the dark color set (DCS) after displaying positive and negative emotional stimuli (*p* < 0.05). After the positive emotional stimuli, participants fixed their sight longer on the light colors (mean value of dwell time = 2112.8 ms), which represents 70% of the given time, to express their positive emotions, while the mean value of the dwell time on the DCS was 525.9 ms, which represents 18% of the given time. On the other hand, after the negative emotional stimuli, participants expressed their negative emotions by focusing on DCS. The mean value of dwell time on DCS was 1669.8 ms (56% of the given time), while it was 906 ms (30% of the given time) on LCS.

The results showed significant differences between genders in dwell time on both LCS and DCS after positive stimuli (*p* < 0.05). Females fixed their sight longer (2556 ms) on the light colors and less (403 ms) on the dark colors than males did (1996 ms, 640 ms on LCS and DCS, respectively) after the positive stimuli. After negative stimuli, a significant difference was found between genders only with dwell time on DCS (*p* < 0.05) where the average dwell time of females‘ eye-fixations (1982 ms) was higher than males’ (1591 ms). 

To investigate the influence of the stimulus’ colors, some of the stimuli were chromatic and others were achromatic. Analysis showed no influence of the stimulus’ color on participants´ eye-fixations between LCS and DCS (*p* > 0.05).

Similarly, the positioning of LCS with respect to the DCS had no significant impact on the participants’ choice (*p* > 0.05). Thus, whether the color scale was graduated (light/dark) from right to left or from left to right, neither had an impact on the participants’ eye-fixations. 

**Figure 9:**
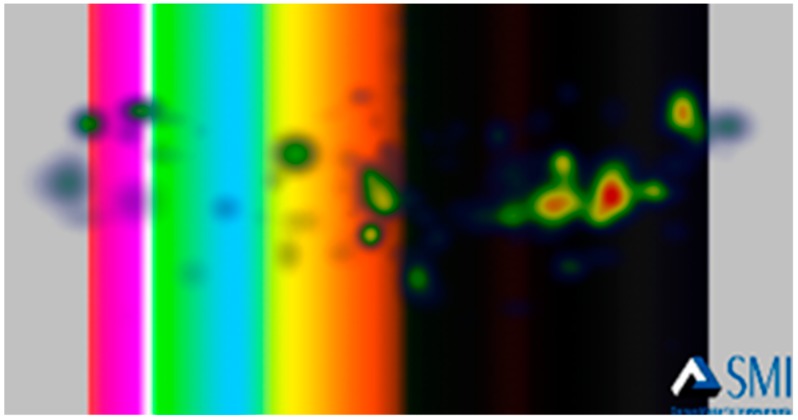
The heat map that demonstrates the eye-gaze patterns over the AOIs after displaying a negative stimulus.

**Figure 10:**
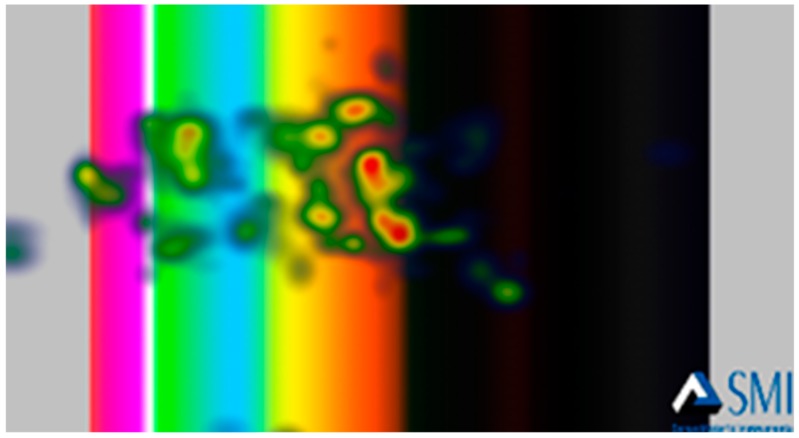
The heat map that demonstrates eye-gaze patterns over the AOIs after displaying a positive stimulus.

Furthermore, heat maps were generated by the BeGaze software to have general insight on the gaze patterns of participants’ eye-fixations. The Heat Map shows how much attention was received at a specific AOI, displayed as colored foci. Figure 9 and Figure 10 show examples of the generated heat maps (focus map) after viewing a negative stimulus and a positive one, respectively. Figure 9 demonstrates higher focused eye-gaze patterns on DCS within 3 s after being shown a negative emotional stimulus. Figure 10 demonstrates the eye-gaze pattern after a positive stimulus.

### 3.3. Sensory Evaluation

A paired sample t-test was conducted for each of the eight samples to investigate how participants used the color scale to express their food-elicited emotion during the sensory evaluation. 

Results showed significant differences in the dwell time (ms) between the eye-fixations on LCS and DCS with all samples except the orange juice bottle and coffee (Table 3). The means of dwell time on LCS for all samples had a higher value than the means of the dwell time on DCS. 

When comparing the participants’ general emotions using VEQ, significant differences were found between GPE and GNE with all samples except for the orange juice bottle. Participants’ rating revealed higher positive emotions than negative emotions toward the samples (Table 3). 

Significant differences between genders were found (*p* < 0.05) with a red bell pepper sample in dwell time on DCS, since males gave a longer dwell time on DCS than females (889.2 ms versus 2190.9 ms, respectively). Although gender had no impact on general emotions regarding red bell pepper samples, results showed that males also had a higher rating for negative emotions than females (mean = 1.2 for females versus 1.5 for males).

## 4. Discussion

To the best of our knowledge, this study is the first to employ emotion-color association together with eye-tracking, as a nonintrusive method, to capture the rapid intuitive eye-fixation on a developed color scale to detect how implicit food-elicited emotions can be expressed using colors. Moreover, the study uses the time pressure conditions as a technique to reduce the conscious impact on the final response and avoid the deliberate reaction.

Our study casts a new light on the emotion-color association and the prospect of using this association to gain insight into implicit emotions.

### 4.1. Emotion-Color Association

Knowing that there is a lack of studies about ECA, this paper first conducted an experiment to investigate how people may use colors to express their emotions. 

The first experiment combines SRQ, as an explicit measure, and eye-tracking, as an implicit measure, to study in-depth both explicit and implicit association between emotions and colors.

The present study confirmed the findings about the strong influence of colors on emotions (the color-emotions association). Light colors evoked highly positive emotions, whereas dark colors made people feel discounted, sad, or evoked other negative emotions. However, the other dimension of the relationship between colors and emotions is also important. If the colors influence our emotions, can we use those colors in return to tell our positive or negative emotions? 

Based on the results of this study, we can conclude that people tend to use light colors to express their positive emotions, and dark colors to express their negative emotions.

Orange, yellow, and green were the most frequently associated colors with the evoked positive emotions, while grey, brown, and black were the most chosen to express the negative evoked emotions. Orange, yellow, and green were reported to be of the greatest frequencies to reveal positive emotions in two previous studies since 1965 [28,29]. This refers to the importance of those three colors in telling positive emotions. Therefore, those three colors were the main colors in the developed color scale. Grey, black, dark green, and dark blue were the most chosen colors after evoking negative emotions. Thus, they were included in the DCS of the developed colors scale as main colors. 

White had very low frequencies with expressing both positive and negative emotions. These low frequencies may have resulted by considering “white” as a neutral color, or the possibility of having different meanings. “White” may evoke the feeling of peace and hop since it is associated with purity and cleanness. On the other hand, it may elicit emotions of emptiness and loneliness [23]. Although red was the fourth in the rank to express positive emotions, it was the fifth chosen color to express negative emotions. Consequently, red and white were excluded from the main colors included in the developed color scale since they may hold a bilateral association. 

SRQ was used as an explicit measure to allow participants to think and choose color samples to express their emotions. On the other hand, eye-tracking, which is a powerful unobtrusive tool, was used as an implicit measure. In addition, literature reports that one of the most important methods to obtain intuitive responses and understand the innate behavior is a time pressure technique [12,45]. Thus, this technique together with eye-tracking were applied in this study to have a better insight on the implicit emotion-color association. 

The eye-tracking results were consistent with SRQ. This is an important finding in the understanding of the ECA. The data showed that the participants chose to focus their eyes on LCS to express their evoked emotions after positive stimuli, while they expressed their negative emotions by focusing their eyes more on DCS. Thus, people can use light and dark colors to reveal their explicit and implicit general emotions. 

Females had a higher dwell time on LCS and lower dwell time on DCS after positive stimuli, in addition to a higher dwell time on DCS after the negative stimuli. This shows that women were faster than men to determine the set of colors that expressed their emotions and fixed their eyes more often on it. Moreover, female participants may tend to link light colors to positive emotions and dark colors to negative emotions more than men do.

Neither the color set’s position (left or right) nor the stimulus’ color (chromatic or achromatic) had an impact on the participant choice. 

This is an important finding for understanding the emotion color association, which will be further employed in a new method.

Since the SRQ and the eye-tracking results were compatible regarding the powerful connection between emotion and colors, the current study takes the advantage of these results to employ the emotion-color association into a new sensory evaluation measure, the color scale, to investigate the implicit food-elicited emotions.

Regarding the food’s characteristics that evoked positive/negative emotions, whenever the food was characterized with the features of tastiness, healthiness, and good environmental impact, it would be the most pleasant food to consume. However, when these three features were not present in one food, “tasty” would be the most important feature that gave people positive feelings. “Only healthy” and “only environmentally-friendly” features had much lower frequencies than “only tasty”. This suggests that, even though people care about health and the environment, the taste of the food plays the leading role when it comes to having a positive emotional food experience.

On the other hand, when the participants described the food that evoked negative emotion, the frequencies of “tasty”, “healthy, but not tasty” and “environmentally-friendly, but not tasty” were rather close. This emphasized the negative emotional impact of the absence of the ‘’tasty” feature in the consumed food. The taste of a healthy or environmentally-friendly food must be, therefore, highly focused on in food processing and marketing in order to encourage consumers to become healthier and more sustainable.

### 4.2. Detecting the Food-Elicited Emotions Using the Developed Color Scale

This part of the study attempts to verify the validity of using the developed color scale in a sensory evaluation. Thus, a VEQ was applied together with the new color scale to investigate the food-elicited emotions.

Our results show a dominance of the positive emotions in the sensory evaluation since the rating of GPE was higher than GNE in all eight samples. A further novel finding is that the current study demonstrates a strong consistency between the VEQ results and the color scale method. In six out of eight samples, significant differences existed between the rating of GPE and GNE and between the dwell time on LCS and DCS. The higher rating of GPE was accompanied with a higher dwell time on the light colors of the color scale to express the positive food-elicited emotions.

Even when no significance results were found, like with the orange juice bottle, red bell pepper, and coffee samples, a higher rating of GPE toward the sample was compatible with a higher dwell time on the light color set to express the food-elicited emotions. The same resemblance was found between the negative emotion ratings and the dwell time on the dark colors.

This consistency highlights the possibility of using colors instead of words as a new tool to express general emotions. Moreover, it may refer to the capability of the developed color scale as a nonintrusive method that avoids any deliberate action and obtains prompt responses to detect rapidly the implicit emotions in a sensory evaluation for a further understanding of consumer’s behavior.

## 5. Limitations and Suggestions for Further Research

Using colors to express the implicit emotions in the present work encourages more avenues for future research on using specific colors to express specific emotions. 

As positive emotions dominate in a sensory evaluation over the negative emotions, an in-depth study presenting food samples that evoke more negative feelings is recommended to use to have better insight on how assessors may use the color scale to express their food-elicited negative emotions. Cross-cultural research on similarities and differences in using the color scale for expressing emotions is also recommended.

## Figures and Tables

**Table 1:** Number, gender percentage, and age of the participants in the experiments.

Experiment		Gender (%)		Age (Years)	Number of Participants
Developing the Color Scale	Self-reported questionnaire	Male	21%	18–88	487
		Female	79%		
	Eye-tracking	Male	37%	18–30	30
		Female	63%		
Sensory Evaluation		Male	35%	19–48	40
		Female	65%		

**Table 2:** The characteristics of the food that evoked positive or negative emotions in the imagined consumption experience.

Food that Evoked	The Features of the Food	Frequency
**Positive emotions**	Healthy/ environmentally-friendly, and tasty	75.0%
	Tasty, even though it was neither healthy nor environmentally-friendly	45.4%
	Healthy, even though it was not tasty	5.0%
	Environmentally-friendly, even though it was not tasty	2.1%
**Negative emotions**	Tasty, but it was neither healthy nor environmentally-friendly	51.4%
	Healthy, but it was not tasty	48.3%
	Environmentally-friendly, but it was not tasty	40.1%

**Table 3:** Mean ratings of general positive and general negative emotions, and mean values of dwell time on the color scale. In addition to the significant differences between GPE and GNE, and dwell time on the light colors versus the dwell time on the dark colors after testing each sample.

Sample	General Positive Emotion	General Negative Emotion	Dwell Time LCS (ms)	Dwell Time DCS (ms)
* ^E, D^ Apple	2.8	1.2	4383.2	827.7
* ^E, D^ Orange juice	2.6	1.4	3303	520.6
* ^E, D^ Walnuts	2.4	1.1	2853.3	1339.4
* ^E, D^ Oregano	2.6	1.4	2825.6	1348.1
* ^E, D^ Red bell pepper	1.9	1.3	3016.6	1344.8
* ^E^ Coffee	2.5	1.3	2363.3	1951.3
Orange juice bottle	1.9	1.7	2746.3	1359.3
* ^E, D^ Pear	2.2	1.2	3486.4	609.9

* Statistically significant. E: General emotion. D: Dwell time.

## Appendix A

Samples of pictures that were used in the negative and positive emotional stimuli.
